# Transcriptomic responses to aluminum stress in tea plant leaves

**DOI:** 10.1038/s41598-021-85393-1

**Published:** 2021-03-11

**Authors:** Danjuan Huang, Ziming Gong, Xun Chen, Hongjuan Wang, Rongrong Tan, Yingxin Mao

**Affiliations:** Institute of Fruit and Tea, Hubei Academy of Agricultural Sciences, Wuhan, China

**Keywords:** Molecular biology, Plant sciences

## Abstract

Tea plant (*Camellia sinensis*) is a well-known Al-accumulating plant, showing a high level of aluminum (Al) tolerance. However, the molecular mechanisms of Al tolerance and accumulation are poorly understood. We carried out transcriptome analysis of tea plant leaves in response to three different Al levels (0, 1, 4 mM, for 7 days). In total, 794, 829 and 585 differentially expressed genes (DEGs) were obtained in 4 mM Al vs. 1 mM Al, 0 Al vs. 1 mM Al, and 4 mM Al vs. 0 Al comparisons, respectively. Analysis of genes related to polysaccharide and cell wall metabolism, detoxification of reactive oxygen species (ROS), cellular transport, and signal transduction were involved in the Al stress response. Furthermore, the transcription factors such as zinc finger, myeloblastosis (MYB), and WRKY played a critical role in transcriptional regulation of genes associated with Al resistance in tea plant. In addition, the genes involved in phenolics biosynthesis and decomposition were overwhelmingly upregulated in the leaves treated with either 0 Al and 4 mM Al stress, indicating they may play an important role in Al tolerance. These results will further help us to understand mechanisms of Al stress and tolerance in tea plants regulated at the transcriptional level.

## Introduction

In neutral or moderately acidic soils, aluminum (Al) is found mainly in the form of insoluble compounds. However, in acidic soil (pH < 4.5), Al tends to solubilize as mononuclear cations (Al^3+^) that are phytotoxic to most plants even at low concentration, which would inhibit root growth, affect the absorption of water and nutrients, and cause a decrease in crop yields^1^. Nevertheless, some plants have adapted to this high Al solubility in acidic soils, especially in tropical regions, by developing resistance or tolerance mechanisms.

Among Al accumulators, the tea plant (*Camellia sinensis* [L.]O. Kuntze) is highly tolerant and adapted to grow on acid soils by sequestering large amounts of Al in the non-toxic forms in its tissues^2^. Al has been reported to be accumulated mainly in tea plant leaves, especially old leaves^3^. Elucidation of the detoxifying strategies of some Al-accumulating plant species such as tea plant may contribute to increasing Al tolerance of crops cultivated in acidic soils. Furthermore, high Al content in tea leaves affects the quality of tea brew^4^, especially in black tea and oolong tea that use mature leaves. Understanding the Al tolerance mechanism in tea plant may lead to reducing uptake and accumulation of Al in leaves and improving tea quality through crop variety improvement^5^.

Previous studies on aluminum in tea plant were mainly about the physiological responses such as mineral element absorption^6–9^, photosynthetic characteristics^10^ and antioxidant defense^11^, as well as the localization and speciation of Al in roots and leaves^12,13^. The main Al tolerance mechanism of tea plant was immobilization of more than 70% of Al in the cell walls. In addition, Al was sequestered in the vacuoles in tea leaves, which could reduce Al toxicity to other organelles^13^. However, our understanding of the molecular mechanisms underlying these processes is still limited. Recently, RNA-Seq has been used to examine the Al-induced alterations of gene expression profiles in plants, including: hydrangea^14^, buckwheat^15^, citrus^16^, maize^17^, and tea plant^18,19^. With this approach, a series of genes possibly responsible for Al-tolerance were identified and characterized, increasing our understanding of the molecular mechanisms of plant Al tolerance. However, most of these studies were focused on the Al-induced alterations of root transcriptome. To our knowledge, little is known about the effects of Al-tolerance on leaf transcriptome. In this study, we used the RNA-Seq to analyze the transcriptome of leaves of tea plants exposed to different concentrations of Al. The aim was to identify the Al responsive genes and gain new insights into molecular mechanisms of Al toxicity and tolerance in tea plants.

## Results

### Leaf Al and pectin contents and antioxidant enzyme activities

**Figure 1:**
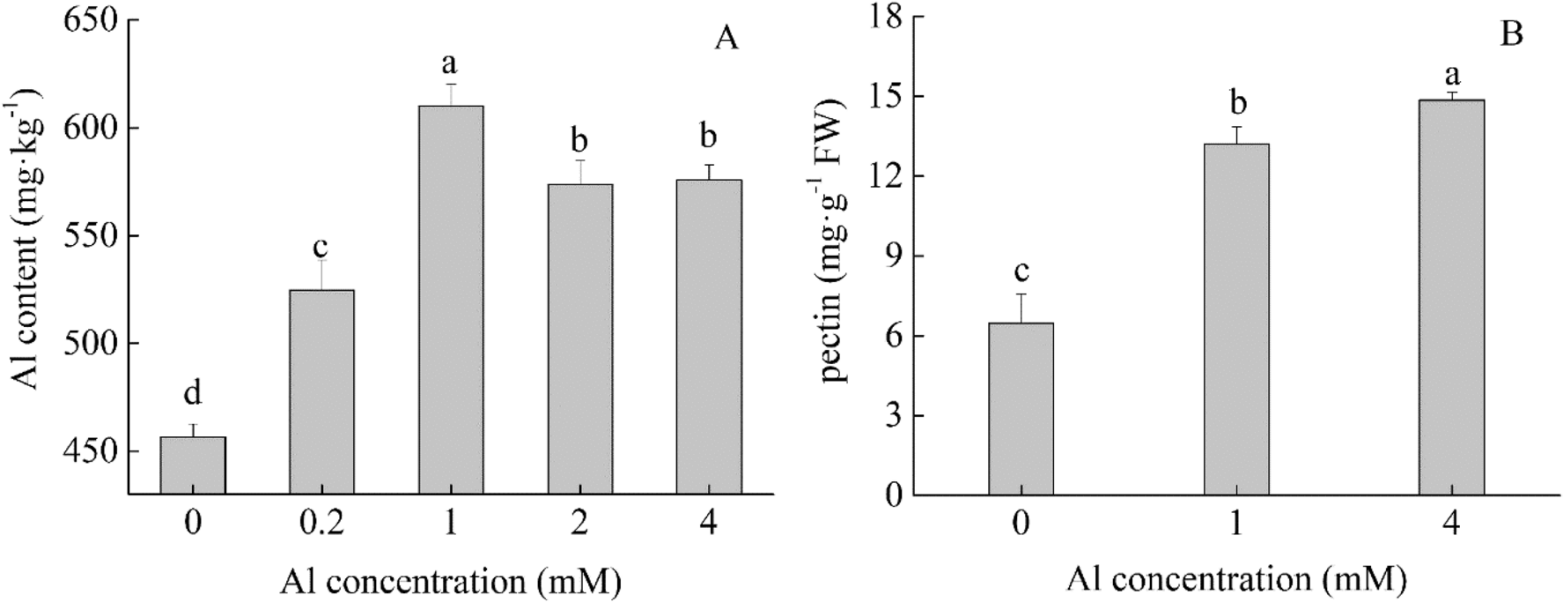
Changes in Al (**A**) and pectin contents (**B**) in tea plant leaves under Al treatment. Values with the same letters are not significantly different (at α = 0.05) from each other.

As shown in Fig. 1, the Al treatment significantly increased Al content in tea plant leaves. The Al content in leaves increased at the tested Al concentration of 1 mM compared with the control, and then decreased at 2 mM.

It had been demonstrated that the cell wall pectin content and Al content were positively related^20^. In the present study, we also found that pectin content and Al content in leaves increased significantly after the Al treatment. Thus, the Al treatment might improve pectin content in tea plant leaves, possibly associated with higher Al concentration in leaves.

**Figure 2:**
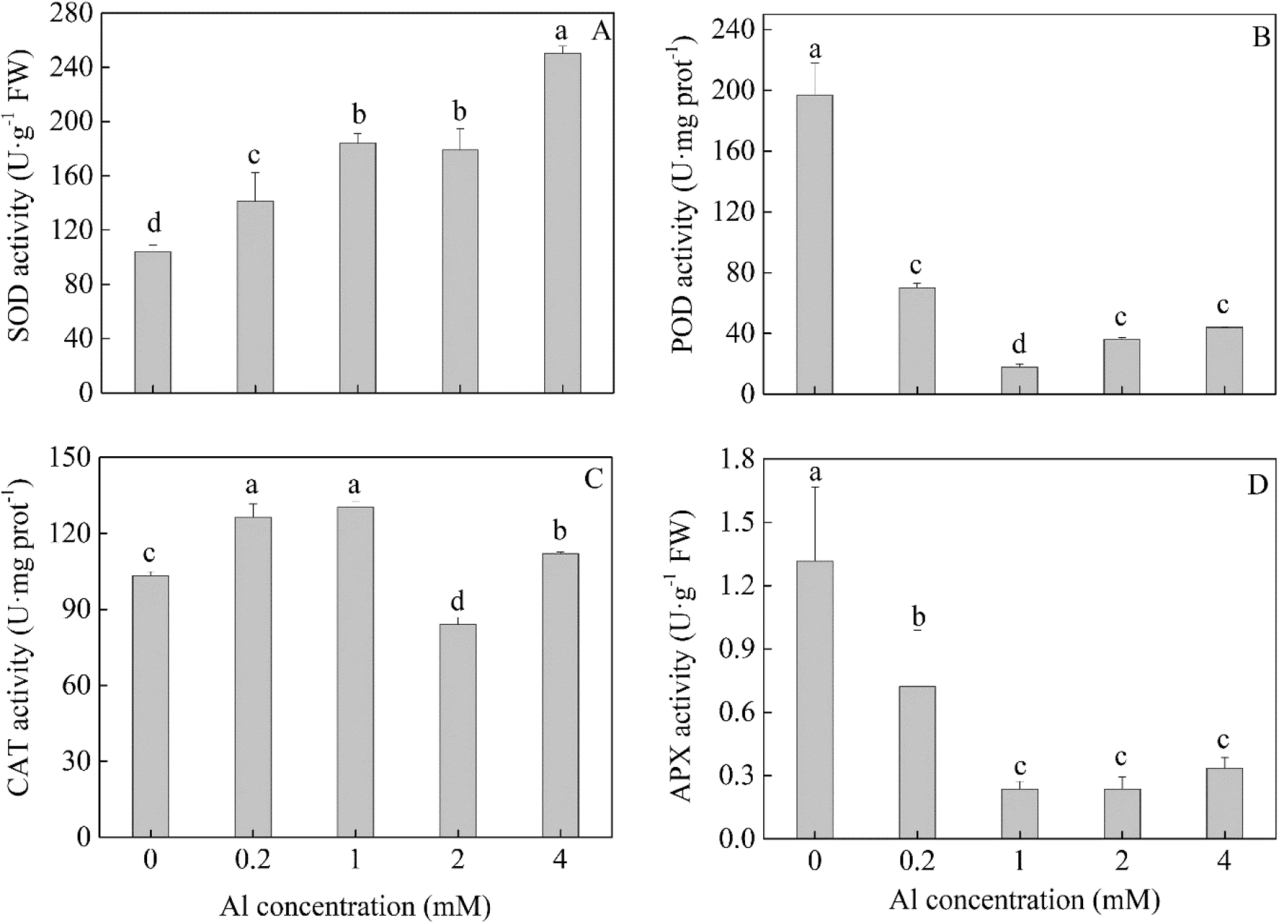
Effect of Al on SOD (**A**), POD (**B**), CAT (**C**), and APX (**D**) activities in tea plant leaves. Values with the same letters are not significantly different (at α = 0.05) from each other.

As for antioxidant enzyme activities (Fig. 2), superoxide dismutase (SOD) activity showed an upward trend with an increase in the Al concentration. Catalase (CAT) activity increased in the Al concentration range of 0–1 mM, but the reverse was the case for peroxidase (POD) and ascorbate peroxidase (APX) activities. The CAT activity at 2 mM was the lowest. For POD and APX activities, there were no significant difference between 2 and 4 mmol L^−1^ Al concentration treatments. The results indicated that different antioxidant enzymes presented various response patterns to Al treatment in tea plant.

### RNA sequencing and reference genome alignment

In total, 41.7 Gb of clean reads were obtained, with an average of 4.6 Gb per sample. The Q20 value for the clean reads was > 98%, and the Q30 value was > 94% (Table S1), indicating the high-quality sequencing results for subsequent analysis. On average, 87.6% of reads were mapped, and the uniformity of the mapping result for each sample suggested they were comparable. The mapping results are shown in Table S2.

### Identification and annotation of differentially expressed genes (DEGs)

**Figure 3:**
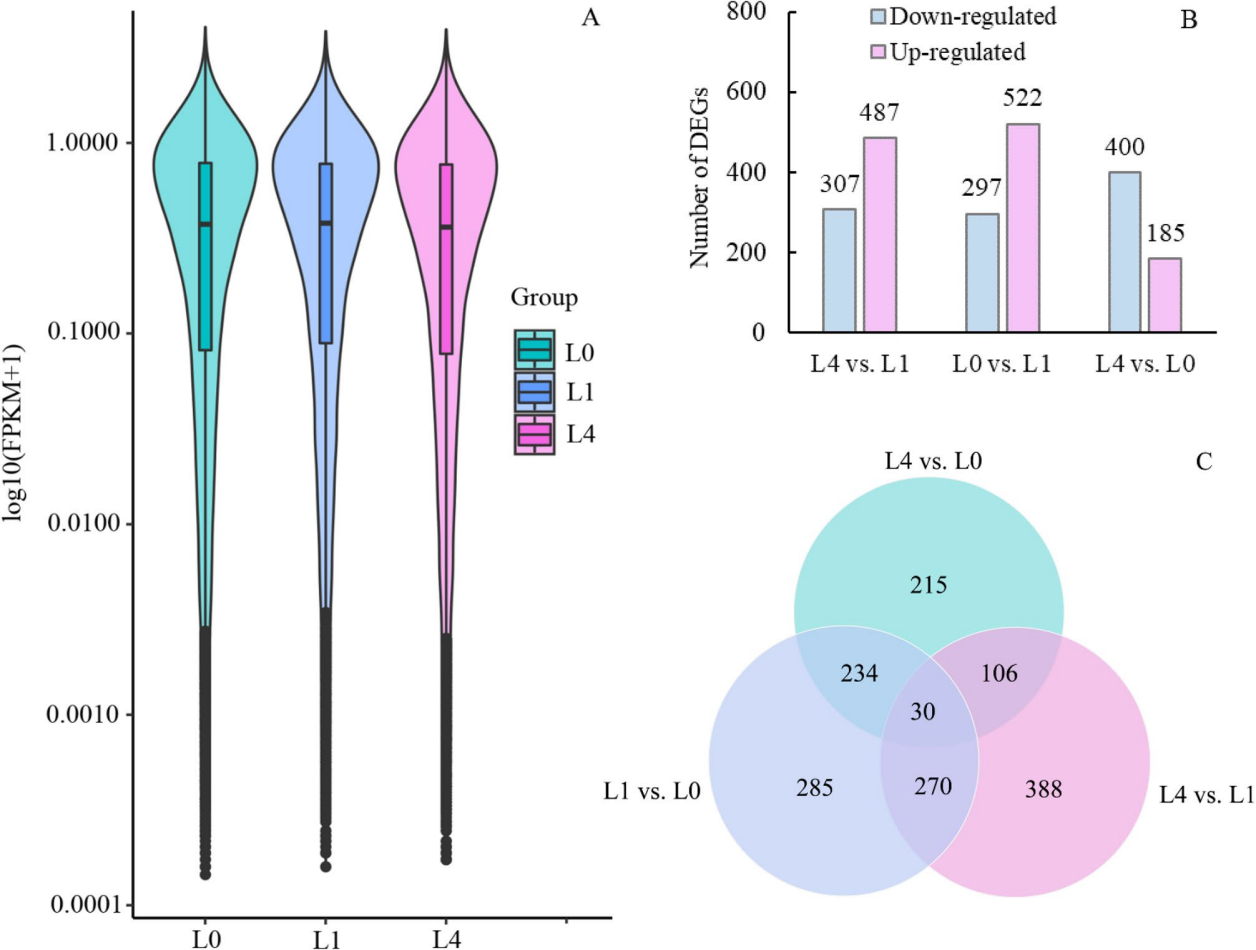
The distribution diagram of gene expression level in L0, L1 and L4 group (**A**), number of DEGs (**B**) and Venn diagram analysis of DEGs (**C**) in the L4 vs. L1, L0 vs. L1, and L4 vs. L0 comparisons.

DEGs were identified by comparing gene expression among 0(L0), 1(L1) and 4(L4) mM. The expression levels were similar in the three treatments were similar, and the log10 (fragments per kilobase per million (FPKM) + 1) values were mainly concentrated around 1(Fig. 3A). In total, 794 DEGs were identified in the comparison L4 vs. L1, with 487 DEGs up-regulated and 307 DEGs down-regulated. Eight hundred twenty-nine DEGs were identified in the comparison L0 vs. L1; of these, 522 DEGs were up-regulated and 297 DEGs were down-regulated. Five hundred eighty-five DEGs were identified in the comparison L4 vs. L0, with 185 DEGs up-regulated and 400 DEGs down-regulated (Fig. 3B). Among all three comparisons above, the total number of down-regulated genes was higher than that of up-regulated genes. Additionally, most DEGs were identified in the L0 vs. L1 comparison group. Only 30 DEGs were identified simultaneously in the three comparisons (Fig. 3C).

**Figure 4:**
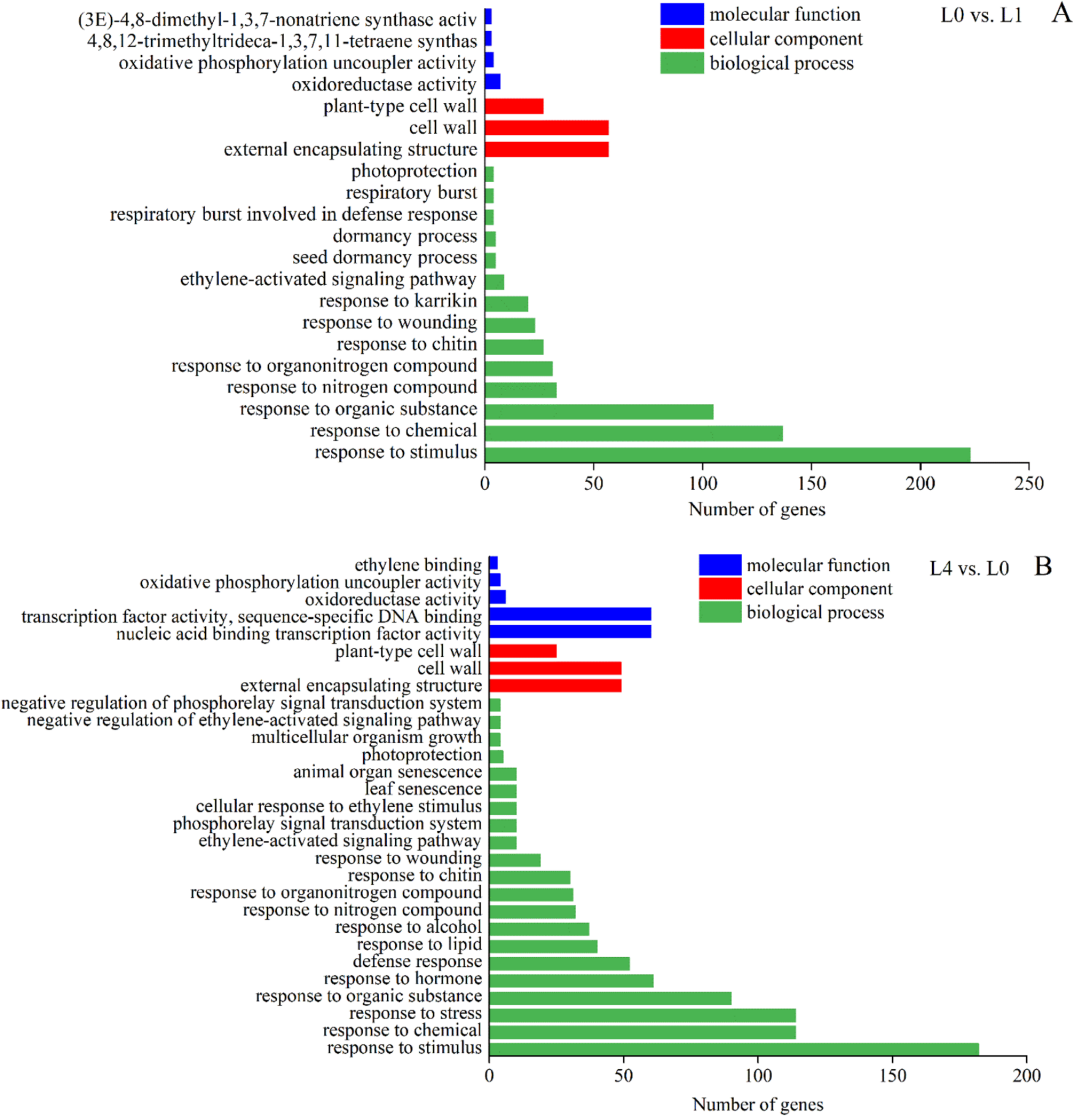
The significant GO enrichment of DEGs. (**A**) L1 vs. L0 comparison, (**B**) L4 vs. L0 comparison. The GO terms associated with biological processes, cellular components, and molecular functions are shown.

The DEGs significantly associated with enriched Gene Ontology (GO) terms and shared by the three comparisons were listed in Fig. 4. The results of GO enrichment analysis showed 21 significantly enriched (false discrimination rate (FDR) ≤ 0.05) GO terms for the L0 vs. L1 comparison and 29 significantly enriched (FDR ≤ 0.05) GO terms for the L4 vs. L0 comparison. For the L0 vs. L1 and L4 vs. L0 comparisons, response to stimulus and cell wall were the top enriched terms in the biological process and cellular component category, respectively. In the molecular function category, oxidoreductase activity was the top enriched term for the L0 vs. L1 comparison, whereas nucleic acid binding transcription factor activity was the top enriched term for the L4 vs. L0 comparison. However, for the L4 vs. L1 comparison, there was no significantly enriched GO term.

**Figure 5:**
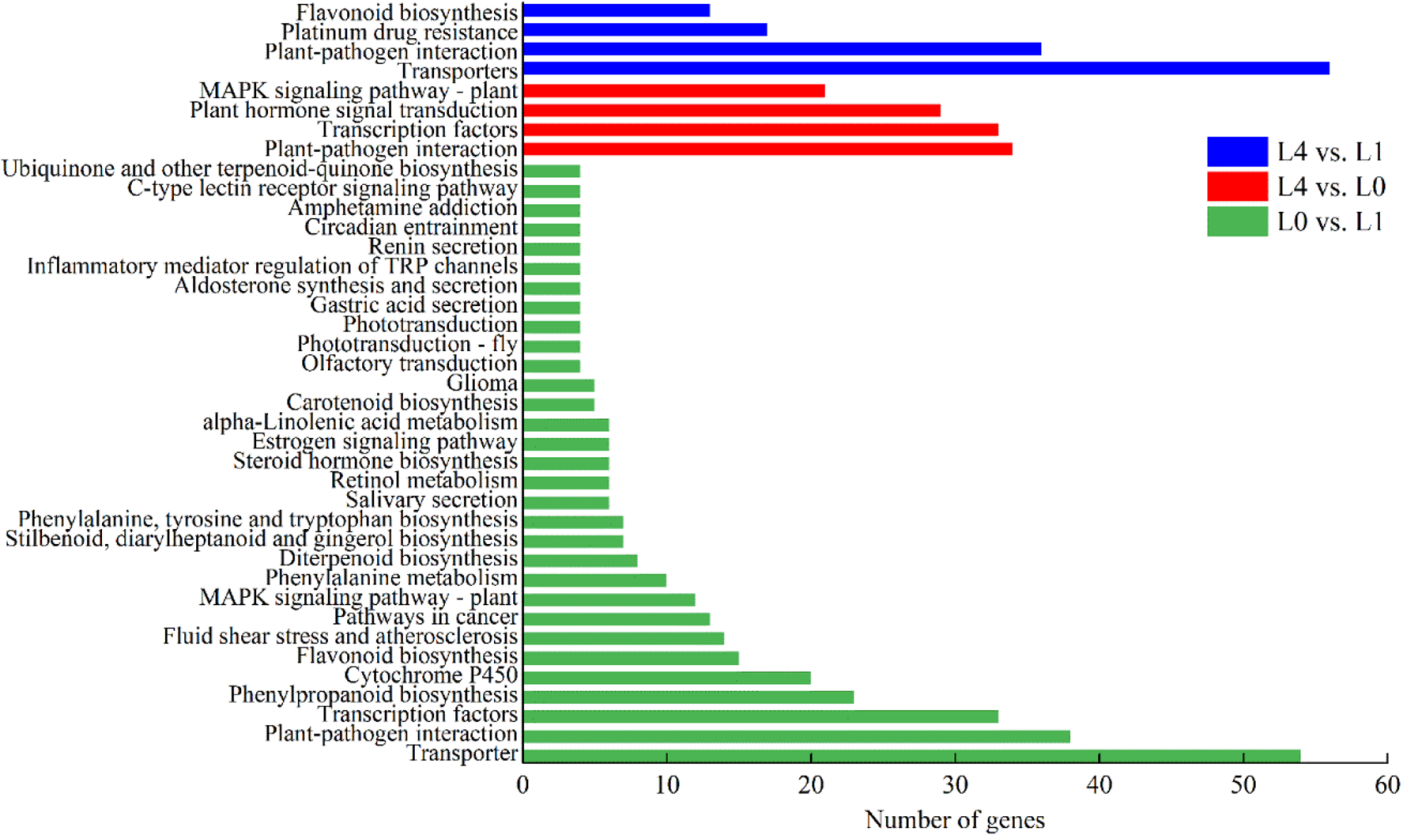
Significantly enriched KEGG enrichment of DEGs from the three different treatment comparisons.

Furthermore, we independently analyzed DEGs in the three Al treatment concentrations to assess whether they were enriched for genes that regulate metabolic pathways (Fig. 5).The DEGs in the L1 vs. L0 comparison were significantly enriched(FDR < 0.05) in 31 pathways. Transporters was the most dominant pathway, followed by plant-pathogen interaction, transcription factors, phenylpropanoid biosynthesis, cytochrome P450, and flavonoid biosynthesis. In the L4 vs. L0 comparison, 4 pathways reached significant levels of enrichment, including plant-pathogen interaction, transcription factors, plant hormone signal transduction, and mitogen-activated protein kinase (MAPK) signaling pathway. In the L4 vs. L1 comparison, the transporters, plant-pathogen interaction and flavonoid biosynthesis pathways were significantly enriched.

### DEGs related to polysaccharide and cell wall metabolism

Plant cell wall consists mainly of polysaccharides (i.e., cellulose, hemicellulose, and pectin), lignin and highly glycosylated proteins^21^. In the present study, regarding polysaccharide and cell wall metabolism, we isolated 5 down-regulated and 7 up-regulated genes in L4 vs. L1, 6 down-regulated and 14 up-regulated in L0 vs. L1, and 9 down-regulated and 4 up-regulated in the L4 vs. L0 comparisons (Table S3). We also isolated four genes encoding cellulose synthases in the L0 vs. L1 and L4 vs. L1 comparisons, indicating these genes showed a same response pattern at the 0 Al as well as the high Al treatments. Hemicellulose, which includes xyloglucan and xylan, was associated with most accumulation of Al in the cell wall of *Arabidopsis*^22^. Here, we isolated two down-regulated xyloglucan endotransglucosylase/hydrolase (XTH) protein genes in the L4 vs. L0 comparison. Furthermore, we identified one up-regulated pectinesterase gene in the L4 leaves, which may play an important role in Al detoxification in tea plant.

### DEGs related to detoxification of ROS

Plants have developed diverse mechanisms for detoxification of ROS elicited by Al in cells^23,24^. A number of genes associated with antioxidative activity and ROS detoxification were identified; most of these genes were up-regulated in the L0 vs. L1 and L4 vs. L1 comparisons, but down-regulated in L4 vs. L0, suggesting that tea plant leaves suffered more oxidative stress in the treatment with 0 Al than high Al (Table S4). Notably, compared with L1, three up-regulated POD genes in L0 and one up-regulated POD gene in L4 were isolated, which were consistent with the changes in POD activities in leaves of L0 and L4 (Fig. 2B).

### DEGs related to cellular transport

Regulated movement of various substances across biological membranes is an important component of cellular stress responses^25^. In this study, we identified all the transporter genes from DEGs in the L4 vs. L1, L0 vs. L1 and L4 vs. L0 comparisons (Table S5), including ABC (ATP-binding cassette) transporter, aquaporin, ion transporter (K, copper, zinc), sugar transporter, amino acid transporters, nitrite transporter, and sulfate transporter. We identified 9 genes encoding ABC transporters (5 up-regulated and 4 down-regulated) in L4 vs. L1, 7 (3 up-regulated and 4 down-regulated) in L0 vs. L1, 5 (4 up-regulated and 1 down-regulated) in the L4 vs. L0 comparisons, indicating that ABC transporters genes were up-regulated with an increase in Al concentration, demonstrating possible involvement of these genes in Al sequestration.

Multidrug and toxin extrusion (MATE) transporters are a large family of membrane transport proteins and can exclude metabolic and xenobiotic organic compounds from the cytosol by exporting them out of the cell or into the vacuole^26,27^. Some members of MATE family can transport citrate that contributed to Al tolerance. Two genes similar to citrate efflux MATE transporter were also found to be down-regulated in tea leaves in the present study. We also found 4 mitochondrial 2-oxoglutarate malate carriers that were responsible for the transport of malate. In contrast, the transporters involved in oxalate were not detected in the present study. Moreover, sulfate transporter and sulfite exporter TauE/SafE were up-regulated in the L0 vs. L1 comparison, indicating an underlying role of these genes in alleviating the sulfite damage of tea leaves in the 0 Al. In addition, nitrite transporter, copper transporter, transmembrane protein 189-like, and sugar carrier protein were up-regulated under Al treatment, although these transporters have not been reported to be associated with Al tolerance; their future investigation will help in further understanding of the mechanism of Al tolerance in tea plants.

### DEGs related to transcription factors (TFs)

The transcription factor genes are very abundant in plant genomes and are represented by a large number of families. Various transcription factors function as the terminal points of stress signal transduction and molecular switches for downstream genes expression^28^. As shown in Table S6, regarding transcription factors, 14 up-regulated and 19 down-regulated genes were detected in L4 vs. L1, 27 up-regulated and 13 down-regulated genes in L0 vs L1, and 10 up-regulated and 23 down-regulated transcription factor genes were identified in the L4 vs. L0 comparison. Most of them belonged to zinc finger, myeloblastosis (MYB), homeobox-leucine zipper protein and WRKY.

### DEGs related to signal transduction

Identification of genes related to signal transduction is helpful for our understanding of the regulation of cellular processes in tea plants by Al. As shown in Table S7, nearly half of these DEGs were found to be involved in phosphorylation and dephosphorylation of proteins, confirming that reversible protein phosphorylation is an important component of Al resistance. Among them, genes encoding serine/threonine protein kinase (STK), receptor-like protein kinase and leucine-rich repeat (LRR) receptor-like serine threonine-protein kinase were identified with down-regulated expression after exposure to high concentration of Al. In addition, DEGs related to hormone biosynthesis degradation, hormone-mediated signal transduction such as abscisic acid (ABA) and indoleacetic acid (IAA), Ca/calmodulin-mediated signal were also found to be differentially regulated in response to Al treatment concentrations.

### DEGs related to phenolics biosynthesis

Phenolics are the most abundant secondary metabolites in tea plants. It has been suggested that some phenolics could detoxify Al in leaves in tea plant^12,29^. In the present study, regarding phenolics biosynthesis and decomposition, we isolated 15 up-regulated and 2 down-regulated genes in L4 vs. L1, and 22 up-regulated and 1 down-regulated genes in the L0 vs. L1 comparison, but only 3 up-regulated and 1 down-regulated genes in the L4 vs. L0 comparison (Table S8). Catechins are a group of flavonoids, accounting for 60% to 80% of the total tea polyphenols. We also found DEGs related to biosynthesis of catechins, such as 4-coumarate-CoA ligase 4, anthocyanidin reductase, flavanone 3-hydroxylase, leucoanthocyanidin reductase, and phenylalanine ammonialyase, to be upregulated under Al stress. These genes may be involved in the internal detoxification of Al by influencing the content of catechins in tea plant leaves.

### Validation of gene expression by qRT-PCR analysis

To validate the results of RNA-seq data, 12 DEGs were selected for qRT-PCR analysis of transcriptional expression. A significant correlation (R^2^ = 0.95, P < 0.05) was observed between the RNA-seq data and the qRT-PCR results (Fig. S1), indicating the high reliability of the RNA-seq data obtained in the present study.

## Discussion

Being an Al-accumulating crop, tea plants display high Al resistance to Al stress; hence, the interaction between Al and tea plant deserves special attention^30^. Al stress is one of the most severe stresses to crops growing in acidic soil. Elucidation of the detoxifying strategies of tea plant may help in breeding genotypes suitable for planting on acid soil. Furthermore, high Al content in tea leaves affects the quality of tea brew, especially in black tea and oolong tea that use mature leaves. In addition, increasing attention is paid to edible tea such as matcha, whereby excessive Al content may affect the safety and thus commercial development of these tea products. Understanding the Al tolerance mechanism of tea plant may lead to reducing uptake and accumulation of Al in leaves, thus improving tea quality and safety. Previous physiological studies have demonstrated that both internal tolerance and external detoxification mechanisms are involved in its high Al resistance. The internal Al tolerance involves Al sequestration in leaves. In the present study, we identified a total of 33,932 genes from tea plant leaves, thus providing a platform for facilitating the future gene function characterization. In addition, 794 DEGs were identified in the L4 vs. L1 comparison, 829 DEGs in L0 vs. L1, and 585 DEGs in L4 vs. L0 after 7 days exposure to Al. These DEGs were functionally categorized into a variety of physiological and molecular responses, which revealed distinct adaptive mechanisms in tea leaves.

Plant cell wall is the major binding site for Al, thus lowering the amount of Al entering to cytoplasm; this was thought to be the crucial exclusion mechanisms in Al detoxification. In tea plants, Al has been reported to be localized mainly in the cell wall of the leaf epidermal cells^31^. In plant cell walls, pectin is a polysaccharide containing galacturonic acids with high electronegativity, which are considered to be the primary binding sites for Al, thus immobilizing it. In the present study, we found that pectin content was increased in leaves with an increase in Al treatment concentration, and one pectinesterase gene was up-regulated in L4 leaves, which might have contributed to the accumulation of Al in leaves. The cell wall architecture can be remodeled for alleviating Al stress by the cell-wall-modifying enzymes^31,32^. For example, many enzymes that mediate the biosynthesis of cell wall polysaccharides are encoded by members of the large cellulose synthase gene superfamily^33^. XTH has been proposed to be responsible for cutting and re-joining inter microfibrillar xyloglucan chains causing cell wall loosening and allowing plant cell expansion^34^. In the present study, we isolated four genes encoding cellulose synthases in the comparison of L0 vs. L1 and L4 vs. L1, implying that cellulose concentration might be changed in these leaves. In addition, we obtained two down-regulated XTH protein genes in the L4 vs. L0 comparison. Given that Al probably did not interact directly with cellulose and hemicellulose, the alterations in these two component were likely to reflect disorderliness of the cell wall metabolism during Al stress.

Antioxidant enzymes are important defense of plants against oxidative stress caused by metals. In the present study, SOD activity increased significantly under Al stress in tea plants, indicating its capacity to scavenge H_2_O_2_. These findings are consistent with the results from the previous studies that showed the activity of SOD was increased in leaves of tea plants by Al exposure^11,35^. Nevertheless, the activity of APX was relatively high at 0 Al, but decreased with increasing Al concentration. As APX activity plays an important role in maintaining the balance between ROS production and elimination, it is likely tea plants showed an effective detoxification of ROS in the absence of Al. A lower activity of POD is beneficial to growth because it plays a role in stiffening the cell wall^36^. We observed that at 1 mM Al, the activity of POD was lowest, which was associated with a stimulatory effect on the growth of tea plants. A decrease in CAT activity was observed at 2 mM compared with 1 mM Al followed by an increase at 4 mM Al. A similar trend was also found in the research of Li et al.^11^. In the present study, we found that many genes potentially involved in ROS production were upregulated by Al stress at the transcriptional level. Collectively, the variability in the expression patterns of antioxidative enzymes suggested a wide range of ROS-related responses to Al in tea plant leaves.

Tea plants accumulate a substantial amount of Al in leaves, suggesting that Al is taken up by roots and translocated to leaves; once in leaf cells, most Al is located in cell walls, and the rest is sequestrated into vacuoles^13^. Thus, a number of different transporters must be involved in these transports. Plant ABC transporters can take up exogenous toxic substances and transport them from cytoplasm into vacuoles for detoxification, consequently protecting the plant cells from damage^37,38^. In the present study, we found that many ABC family transporters genes were up-regulated with an increase in Al concentration, implying their important roles in Al detoxification in leaf vacuole.

Al tolerance also involves internal mechanisms that allow tea plants to detoxify Al by forming nontoxic Al complexes in the cytosol. It has been reported that Al occurred primarily as Al–catechin complexes in tea leaves^39,40^, whereas Al–citrate complexes occurred in the xylem sap and Al–oxalate complexes in the roots of tea plants^41,42^. In the present study, we found that the transporters involved in citrate and malate were downregulated in leaves under Al treatment, and transporters involved in oxalate were not detected in the present study. Evidence presented thus far did not support the idea these three organic acids were the primary Al complexing compounds in leaves. In contrast, genes involved in phenolics biosynthesis and decomposition were mostly up-regulated in L4 compared with L1. This has led us to speculate that phenolics might be the primary Al-complexing compounds in leaves. In addition, nitrite transporter, zinc transporter, copper transporter, transmembrane protein 189-like, sugar carrier protein were up-regulated in the Al treatment; these genes may regulate and maintain cell homeostasis by affecting the transport of metal ions, amino acids and osmotic substances.

A number of transcription factors including zinc finger protein, basic leucine zipper (bZIP), WRKY, MYB, GATA, NAC, bHLH25-like, and heat shock transcription factor, were detected in the present study, some of which may be involved in Al resistance. Zinc finger protein genes play a significant role in plant stress responses^43^. For example, the first transcription factors to be identified as involved in regulation of Al resistance in plants, sensitive to proton rhizotoxicity 1(STOP1)/ Al resistance transcription factor 1 (ART1), are the kind of C2H2-type zinc finger transcription factors^44,45^. In the present study, we identified a downregulated C2H2-type zinc finger transcription factor. Further functional analysis will reveal its roles in Al detoxification and accumulation in tea plants.

MYB transcription factors have been found to be involved in a variety biotic and abiotic stresses^46^. Previous studies documented the expression of *OsMYB2P-1* was induced by P deficiency, and transgenic *Arabidopsis* and rice plants overexpressing *OsMYB2P-1* had enhanced tolerance to P-deficiency^47^. In the present study, we identified five upregulated MYBs in the L4 vs. L1 comparison. Thus, the Al-induced upregulation of MYB transcription factors might contribute to Al tolerance of tea plants via enhancing tolerance to P starvation. However, we also isolated several transcription factors upregulated in the L0 vs. L1 comparison, including WRKY, NAC and bHLH25-like transcription factors, indicating that these transcription factors were repressed by Al. The exact roles of these transcription factors need to be elucidated in further investigation. In conclusion, the large differences in the Al-induced alterations of TF expression profiles implied that TFs might play a role in Al-tolerance of tea plants.

## Conclusion

Through RNA-seq data analysis, we isolated 794, 829 and 585 Al-responsive genes in the comparisons L4 vs. L1, L0 vs. L1 and L4 vs. L0, respectively. Through the integration of the present findings and the available data in the literature, the common and unique mechanisms of Al tolerance in tea plants were proposed. The following aspects might account for the high Al accumulation in leaves of tea plants: (a) Tea plants displayed an efficient internal mechanism of binding Al to the cell wall and transport excess Al to vacuole, (b) tea plants exhibited an efficient chelation system through forming stable Al-catechin complexes, and (c) the quick signal transduction and high antioxidant capacity reduced the Al-related damage to tea plant cells. Collectively, transcriptome results provided clues that more attention should be paid to pectinesterase, ABC transporter, MATE transporter and genes involved in catechin biosynthesis. Although the exact roles of these candidate genes remain to be elucidated, the results of this study provide a platform for further functional analysis of these genes.

## Methods

### Plant material and treatments

Two-year-old tea plants (cv. ‘Fuding Dabaicha’) were grown in the Hubei Academy of Agricultural Sciences, Wuhan, China. The rooted cuttings were washed and transplanted to nutrient solutions containing the macronutrients (mM): NH_4_NO_3_ (1.0), KH_2_PO_4_ (0.07), K_2_SO_4_ (0.3), MgSO_4_ (0.67), CaCl_2_ (0.53), and Al_2_(SO_4_)_3_ (0.07) and micronutrients (µM) Fe-EDTA (4.2), H_3_BO_3_ (7), MnSO_4_ (1), ZnSO_4_ (0.67), CuSO_4_(0.13), and (NH_4_)_6_Mo_7_O_24_ (0.05). The pH of the solution was adjusted to 5.0 with 1 mM NaOH or H_2_SO_4_. The plants were cultivated in a greenhouse under a day/night ratio of 16/8 h. Relative humidity and temperature were kept at 65–85% and 22–25 °C. The solutions were and refreshed every week until the first trifoliate leaves expanded. Then, different Al treatments were (0, 0.2, 1, 2, and 4 mM) were imposed, with three replicates of each treatment. After one week, the third leaves in each treatment were harvested, snap frozen in liquid nitrogen and stored at − 80 °C until further use.

### Antioxidant enzyme activities, pectin content and Al determination

The activities of SOD, POD, CAT and APX were determined by using the reagent kits (Jiancheng, Nanjing, China) according to the manufacturer’s instruction. The pectin content was determined by using the reagent kit (Yuduo, Shanghai, China) according to a manufacturer’s instructions. An amount of 0.4 g fresh weight of leaf sample powder was digested in a mixture of HNO_3_:HClO_4_ (5:2; v/v), adjusted to 50 mL with distilled water, and filtered through a 0.45-μm organic membrane before Al was analyzed by inductively coupled plasma optical emission spectrometer (ICP-OES). The measurements were repeated thrice, and the average values ± standard error were calculated.

### RNA extraction, library construction, Illumine sequencing, and genome mapping

Leaf samples from the treatments with Al concentration of 0 (L0), 1 (L1) and 4 (L4) mM were collected for RNA-Seq analysis. The Al concentration at 1 mM, was used as the control (CK) because of good root growth at that concentration. Equal amounts of frozen leaves from three individual plants were mixed as one biological replicate. There were three biological replicates for each treatment. Total RNA was extracted using an RNeasy Plant Mini Kit (TaKaRa, Dalian, China), according to the manufacturer’s protocol. The concentration and quality of total RNA were examined using a NanoDrop 2000c spectrophotometer (NanoDrop, Wilmington, DE, USA) and 1% w/v agarose gel electrophoresis. The cDNA libraries were sequenced by a llumina Hiseq Xten platform by the Bioacme Biotechnology Co., Ltd. (Wuhan, China). The high-quality clean reads from each sample were mapped to the reference genome^48^ using Hisat2.

### Differential expression analysis, GO and KEGG enrichment analysis

Gene expression levels were calculated using the fragments per kilobase million (FPKM) method^49^. Differentially expressed genes (DEGs) among the three comparisons (L4 vs. L1, L0 vs. L1, and L4 vs. L0) were identified using DESeq^50^. A false discovery rate of ≤ 0.05 and an absolute fold-change value ≥ 1 were used as the criteria for identifying DEGs. GO enrichment analysis of differentially expressed genes was implemented by the GOseq R package, in which gene length bias was corrected. The GO terms with corrected P value less than 0.05 were considered significantly enriched for differentially expressed genes. The cluster profiler package in R language was used to conduct a KEGG (Kyoto Encyclopedia of Genes and Genomes) enrichment analysis, and the DEGs involved in metabolic pathways were determined^51^.

### Validation of RNA-seq by quantitative Real time RT-PCR

Analysis of qRT-PCR was performed using a SYBR Green PCR Mix (Simgen, Hangzhou, China). *CsGAPDH* was used as the internal reference gene. Real-time qPCR was performed on an Applied Biosystem 7500 Real-Time PCR system (Thermo Fisher Scientific, USA) using the following program: 95 °C for 30 s ; 40 cycles of 95 °C for 5 s each, annealing at 60 °C for 34 s; 95 °C for 15 s, 60 °C for 1 min, and 95 °C for 15 s. The 2^−ΔΔCt^ method^52^ was employed to calculate the relative expression. The specific primer sequences are shown in Table S1. All measured expression levels were determined in six replicates and the results were presented as mean ± SD.

## Supplementary information

Supplementary information.
