# Supplementary material for: Transcriptomic responses to aluminum stress in tea plant leaves
Source: Sci Rep. 2021 Mar 11;11:5800. doi: 10.1038/s41598-021-85393-1 (PMC7952733; doi:10.1038/s41598-021-85393-1)
Supplement: Supplementary file 1 — Supplementary information. [file 41598_2021_85393_MOESM1_ESM.pdf]

Transcriptomic responses to aluminum stress in tea plant leaves

Danjuan Huang, Ziming Gong, Xun Chen, Hongjuan Wang, Rongrong Tan, Yingxin Mao*
Institute of Fruit and Tea, Hubei Academy of Agricultural Sciences, Wuhan, China;
* e-mail: maoyingxin@126.com

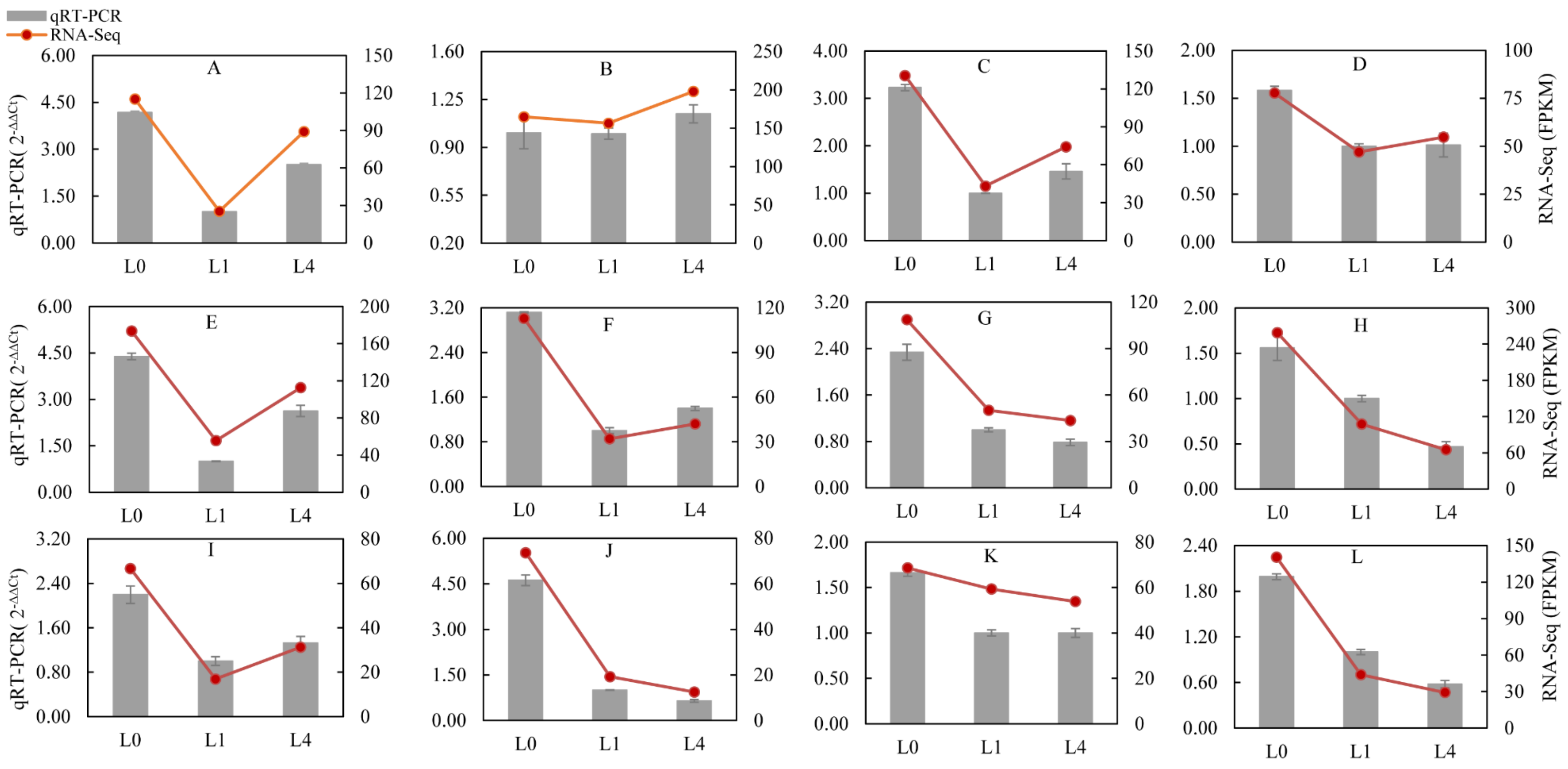

Figure S1. Gene expression validation by qRT-PCR. The red lines (referring to right axis) stand for the gene expression in RNA-Seq analysis and gray bars (referring to left axis) stand for the qRT-PCR result. (A) flavanone 3-hydroxylase. (B) Auxin responsive protein. (C) Galactinol--sucrose galactosyltransferase. (D) AAA-type ATPase family protein. (E) anthocyanidin reductase. (F) Mitochondrial 2-oxoglutarate malate carrier. (G) calcium-binding protein. (H) CCR4-associated factor. (I) Aquaporin. (J) Mitochondrial 2-oxoglutarate malate carrier. (K) Glutathione S-transferase. (L) EF hand family protein.

Table S1. Summary of the RNA-Seq data collected from Al-treated and control leaves of *C. sinensis*. Q20 and Q30 mean sequencing error rates lower than 1% and 1‰, respectively.

Samples	Raw reads	Clean reads(%)	Clean bases(G)	%>=Q20	%>=Q30	GC(%)	Mean Quality Score
L0_1	26354391	24655947(93.56)	3.44	98.17	94.86	43.95	38.68
L0_2	38890513	37686372(96.90)	5.47	98.50	94.79	44.48	36.25
L0_3	43084681	41543408(96.42)	6.06	98.49	94.69	44.42	36.23
L1_1	21269955	19842168(93.29)	2.79	98.39	95.25	43.91	38.70
L1_2	34838224	33998207(97.59)	4.97	98.83	95.80	44.15	36.41
L1_3	31231662	30442215(97.47)	4.45	98.77	95.60	43.68	36.38
L4_1	30589869	28276195(92.44)	3.97	98.60	95.87	43.98	38.66
L4_2	33610411	32696575(97.28)	4.78	98.72	95.42	44.47	36.34
L4_3	41388798	40067747(96.81)	5.79	98.54	95.04	45.02	36.30

Table S2. Summary of clean reads and genes mapped to the reference genome from Al-treated and control leaves of *C. sinensis*.

SampleID	Input reads	Mapped reads	Mapped rate %	non-unique	unique	unique Mapped rate%	Read-1	Read-2	Concordant pair alignment rate
L0_1	49311894	43019041	87.24	5411420	37607621	87.42	18825253	18782368	80.06%
L0_2	75372744	65915354	87.45	11003219	54912135	83.31	27524160	27387975	81.68%
L0_3	83086816	72255010	86.96	13297998	58957012	81.60	29557254	29399758	79.98%
L1_1	39684336	34633318	87.27	4400401	30232917	87.29	15130041	15102876	79.53%
L1_2	67996414	59698132	87.80	10650556	49047576	82.16	24602653	24444923	79.80%
L1_3	60884430	53468326	87.82	8763713	44704613	83.61	22396953	22307660	80.03%
L4_1	56552390	49534805	87.59	6463189	43071616	86.95	21539713	21531903	79.26%
L4_2	65393150	57536105	87.98	9454835	48081270	83.57	24064442	24016828	80.30%
L4_3	80135494	71019660	88.62	12702530	58317130	82.11	29231639	29085491	83.18%

Table S3. DEGs related to polysaccharide and cell wall metabolism.

Gene ID	Description	L4 VS L1		L0 VS L1		L4 VS L0	
		log2(FC)	FDR	log2(FC)	FDR	log2(FC)	FDR
TEA033374.1	alpha 1,4-glycosyltransferase family protein			1.13	4.411E-06		
TEA004596.1	beta-1,4-glucan synthase					-1.49	2.51E-05
TEA026003.1	Bifunctional monodehydroascorbate reductase and carbonic anhydrase					-2.38	0.0003017
TEA003176.1	cellulose synthase-like protein	-2.47	3.223E-07	-1.68	0.0003592		
TEA003407.1	cellulose synthase-like protein G1-like	-2.88	0.0021876	-3.44	2.06E-11		
TEA003410.1	cellulose synthase-like protein G1-like	2.14	6.161E-11	1.63	2.708E-05		
TEA031419.1	cellulose synthase-like protein G1-like	1.92	1.108E-12	1.69	1.132E-08		
TEA019135.1	chitinase			1.41	5.851E-09	-1.46	1.194E-09
TEA006711.1	Cinnamyl alcohol dehydrogenase			1.24	4.368E-06		
TEA024897.1	Cinnamyl alcohol dehydrogenase			1.10	4.691E-11		
TEA025404.1	Cinnamyl alcohol dehydrogenase			1.11	0.0034455		
TEA005738.1	Endoglucanase					-1.52	0.0009259
TEA008777.1	Exostosin family			-3.02	0.0140777		
TEA028320.1	exostosin family	-1.06	1.977E-10				
TEA014210.1	expansin-like	1.03	6.115E-06				
TEA010582.1	Galacturonosyltransferase			-1.12	0.0375801		
TEA004682.1	Galacturonosyltransferase-like			-1.03	0.0337881		
TEA006330.1	Galacturonosyltransferase-like			-3.95	1.461E-09	-4.95	4.382E-14
TEA028187.1	galacturonosyltransferase-like 9-like					-1.29	0.0001094
TEA029798.1	glucan endo-1,3-beta-glucosidase			0.00		1.01	0.001972
TEA000108.1	glucan endo-1-3-beta-glucosidase			1.20	6.968E-13		
TEA019132.1	glucan endo-1-3-beta-glucosidase	1.12	1.747E-05				
TEA030572.1	glucan endo-1-3-beta-glucosidase			0.00		-1.29	4.132E-05
TEA020331.1	glucuronoxylan 4-O-methyltransferase activity	-2.32	0.000748	-1.78	0.0134859		
TEA014600.1	Glycerophosphodiester phosphodiesterase	1.97	4.975E-15	1.84	4.672E-09		
TEA018449.1	inositol oxygenase	2.01	8.683E-70	1.46	3.327E-44		
TEA015181.1	invertase pectin methylesterase inhibitor family protein DC			-1.02	2.251E-22	1.85	3.911E-37
TEA004576.1	pectinesterase	1.29	0.0125303			1.15	0.0192768

TEA015891.1	xyloglucan			-1.33	0.0164792	1.22	0.0375719
TEA003768.1	Xyloglucan endotransglucosylase hydrolase protein	-1.51	2.066E-19			-1.56	1.022E-10
TEA023101.1	Xyloglucan endotransglucosylase hydrolase protein			1.07	0.0041099	-1.11	0.0016312

FC, fold change; FDR, false discovery rate.

Table S4. DEGs related to detoxification of ROS.

GeneID	Description	L0 VS L1		L4 VS L0		L4 VS L1	
		log2(FC)	FDR	log2(FC)	FDR	log2(FC)	FDR
TEA000474.1	2OG-Fe(II) oxygenase superfamily	1.567	3.65E-16			1.94	8.86918E-28
TEA009011.1	2OG-Fe(II) oxygenase superfamily	1.394	0.000131142			1.37	5.39832E-09
TEA031172.1	2OG-Fe(II) oxygenase superfamily	2.557	8.10E-10			1.67	0.001897837
TEA031175.1	2OG-Fe(II) oxygenase superfamily	2.663	1.09E-07			1.42	0.044487282
TEA004906.1	2-oxoglutarate Fe(II)-dependent dioxygenase-like					1.22	4.46237E-10
TEA007101.1	2-oxoisovalerate dehydrogenase subunit alpha	1.904	1.04E-06			2.10	2.70225E-09
TEA019049.1	3-hydroxy-3-methylglutaryl-coenzyme A reductase					1.42	1.45632E-06
TEA028769.1	3-hydroxy-3-methylglutaryl-coenzyme A reductase	1.327	3.99E-15			1.18	1.87422E-13
TEA031782.1	3-ketoacyl-coa synthase					-1.21	0.021377085
TEA015906.1	3-ketoacyl-coa thiolase	1.200	8.14E-10				
TEA022237.1	9-cis-epoxy-carotenoid dioxygenase			-1.26	9.94215E-07	-1.02	7.97211E-06
TEA025155.1	9-cis-epoxy-carotenoid dioxygenase	1.102	0.001637415				
TEA030362.1	9-cis-epoxy-carotenoid dioxygenase	1.518	0.023379882				
TEA029408.1	AAA-type ATPase family protein	1.363	0.00112404				
TEA029314.1	alcohol dehydrogenase	1.147	4.81E-05			1.42	0.001758285
TEA001041.1	Allene oxide synthase					1.85	0.015378177
TEA020533.1	Bifunctional monodehydroascorbate reductase and carbonic anhydrase	-3.524	2.21E-06			-3.02	5.88831E-06
TEA028163.1	cytochrome	1.295	8.75E-10			1.09	1.01675E-10
TEA000599.1	cytochrome P450	-5.896	3.52E-08			-6.47	3.57165E-07
TEA001544.1	cytochrome P450			-1.00	3.21309E-07		
TEA002351.1	cytochrome P450	1.345	0.016326235	-1.59	0.002392285		
TEA004534.1	cytochrome P450					1.72	0.000356322
TEA007092.1	cytochrome P450	-1.720	0.02511584			-2.03	0.002379778
TEA010986.1	cytochrome P450	1.496	0.002034594				

TEA010992.1	cytochrome P450			-2.69	3.52384E-35		
TEA013801.1	cytochrome P450	1.862	3.40E-23			2.60	6.16359E-52
TEA014790.1	Cytochrome p450					1.53	0.040134075
TEA017695.1	Cytochrome p450	3.079	0.004892509			3.73	4.39346E-05
TEA018271.1	cytochrome P450	2.718	1.86E-23			3.52	2.41935E-49
TEA019521.1	cytochrome P450	1.588	0.000623628				
TEA022686.1	cytochrome P450			-1.24	3.1163E-09		
TEA022705.1	cytochrome P450			-1.06	0.022818074		
TEA025560.1	cytochrome P450			1.23	2.11237E-12	1.72	1.42659E-27
TEA025967.1	cytochrome P450					-5.06	3.75297E-07
TEA025969.1	cytochrome P450	-6.591	0.00011084			-7.53	0.002732895
TEA026170.1	cytochrome P450					1.17	3.68304E-12
TEA028023.1	cytochrome P450	2.495	2.74E-31			2.72	1.7748E-39
TEA029768.1	cytochrome P450			-1.22	1.55285E-05		
TEA030439.1	cytochrome P450	-1.273	0.000122342				
TEA032382.1	Cytochrome p450	-1.178	1.44E-05				
TEA032825.1	cytochrome P450	2.148	0.027184795			2.48	0.003858449
TEA033367.1	epoxide hydrolase					1.17	0.0054108
TEA016604.1	FAD binding domain	-6.371	0.000302381				
TEA027908.1	FAD binding domain					-1.01	0.008608816
TEA004752.1	galactinol synthase	1.339	6.85E-09	-1.04	4.00553E-05		
TEA006802.1	galactinol synthase	1.279	0.011063629	-1.04	0.033409168		
TEA000817.1	germin-like protein			1.24	1.15392E-06	1.03	1.79373E-05
TEA010583.1	germin-like protein	1.789	0.000472438				
TEA011002.1	germin-like protein					-1.38	0.013736282
TEA014855.1	germin-like protein	2.978	0.033296618	-2.63	0.039479202		
TEA016870.1	germin-like protein	1.254	2.17E-07				
TEA016871.1	germin-like protein	1.287	1.82E-08				
TEA018757.1	germin-like protein			-1.85	0.030279694		

TEA023353.1	germin-like protein	1.818	0.015872843				
TEA024914.1	germin-like protein	2.057	0.004675132	-2.17	0.001707102		
TEA027597.1	germin-like protein	1.520	0.000123552				
TEA029053.1	germin-like protein	2.121	0.04249811				
TEA012816.1	glutaredoxin-C9-like	1.154	1.58E-11				
TEA026750.1	glutaredoxin-C9-like	1.210	2.58E-09				
TEA010114.1	glutathione s-transferase	2.613	0.014860475			2.89	4.10053E-05
TEA022693.1	Glutathione S-transferase	2.722	1.03E-06			2.72	6.14877E-08
TEA025564.1	Glutathione S-transferase	1.726	0.000253866			2.41	4.87925E-10
TEA025571.1	Glutathione S-transferase					1.28	0.012908504
TEA030910.1	hydroxycinnamoyl-Coenzyme A shikimate quinate hydroxycinnamoyltransferase-like	-4.736	2.11E-08			-5.88	5.25127E-08
TEA006446.1	Inherit from COG: fad dependent oxidoreductase	-1.007	0.012914839				
TEA018449.1	inositol oxygenase	1.460	3.33E-44				
TEA014279.1	long-chain-alcohol O-fatty-acyltransferase			1.21	2.30574E-07		
TEA022425.1	Long-chain-alcohol oxidase	-1.300	5.40E-11			-1.03	2.56894E-07
TEA006738.1	Multicopper oxidase	-2.411	0.000251575			-1.92	0.000751919
TEA031514.1	NADH dehydrogenase)-like	1.053	0.000302381			1.15	2.01463E-09
TEA026453.1	NADH-Ubiquinone oxidoreductase	1.581	0.04249811				
TEA004042.1	oxidoreductase transition metal ion binding protein					-1.02	0.012960438
TEA002405.1	oxidoreductase, 2OG-Fe(II) oxygenase family protein					1.67	2.39686E-05
TEA021784.1	oxidoreductase, 2OG-Fe(II) oxygenase family protein	1.631	0.012666112				
TEA011393.1	peroxidase					1.53	5.51125E-17
TEA015018.1	peroxidase					1.05	1.04112E-05
TEA015805.1	peroxidase	1.084	0.000448511				
TEA026798.1	peroxidase	1.560	4.11E-05	-1.33	0.00016371		
TEA028696.1	peroxidase	1.086	0.020377013				
TEA029796.1	peroxidase					-1.34	0.001102542
TEA025746.1	protein disulfide oxidoreductase activity			-1.00	8.10055E-05		

TEA015549.1	Quinone oxidoreductase-like protein At1g23740					1.06	0.010683833
TEA000024.1	respiratory burst oxidase	1.042	7.26E-06			1.32	6.68793E-10
TEA028693.1	respiratory burst oxidase	1.072	0.040123756				
TEA010579.1	reticuline oxidase-like protein-like	1.583	0.00753029	-1.29	0.026162613		
TEA020054.1	reticuline oxidase-like protein-like			-1.13	2.21659E-09		
TEA004730.1	Ribulose bisphosphate carboxylase oxygenase activase			-1.13	8.04762E-08		
TEA016417.1	shikimate quinate hydroxycinnamoyltransferase-like					-1.20	5.48194E-05
TEA030909.1	shikimate quinate hydroxycinnamoyltransferase-like					-5.73	0.00056183
TEA025563.1	Thioredoxin	1.967	1.07E-12	-1.04	0.000475433		
TEA014719.1	Ubiquinol oxidase			-1.85	0.007053302		

FC, fold change; FDR, false discovery rate.

Table S5. DEGs related to cellular transport.

geneID	Description	L4 VS L1		L0 VS L1		L4 VS L0	
		log2(FC)	FDR	log2(FC)	FDR	log2(FC)	FDR
TEA001106.1	ABC transporter			1.72	3.34E-07		
TEA018843.1	ABC transporter	1.36	0.0168178			1.23	0.034142743
TEA031499.1	ABC transporter	-1.42	4.611E-05	-2.26	4.11E-08		
TEA005016.1	ABC transporter B family member	1.23	0.0052459				
TEA014428.1	ABC transporter B family member	1.23	4.291E-07			1.10	2.71794E-06
TEA003914.1	ABC transporter C family member			-2.80	0.0001235	2.05	0.036673649
TEA003916.1	ABC transporter C family member			-3.07	0.0008343		
TEA008884.1	ABC transporter C family member	-1.03	1.551E-06				
TEA008891.1	ABC transporter C family member	-1.17	0.0006077				
TEA016647.1	ABC transporter C family member	-1.18	0.0001903	-1.07	1.48E-05		
TEA015812.1	ABC transporter D family member	1.85	3.226E-26	1.33	1.63E-11		
TEA005434.1	ABC transporter G family member			1.13	3.03E-18	-1.54	5.56317E-37
TEA010703.1	ABC transporter I family member	1.35	3.2E-08			1.25	1.15392E-06
TEA000981.1	amino acid transporter			1.31	9.29E-05	-1.26	7.13831E-05
TEA011399.1	amino acid transporter			1.18	0.0003907		
TEA015946.1	anion transporter	1.67	3.972E-07	2.35	3.20E-07		
TEA008965.1	Aquaporin			1.37	4.25E-16		
TEA012255.1	Aquaporin			2.18	0.0051704		
TEA013356.1	Aquaporin			1.52	2.99E-14		
TEA013439.1	Aquaporin	2.51	3.292E-18			1.75	3.11455E-10
TEA019952.1	Aquaporin			1.76	6.11E-06		
TEA027942.1	Aquaporin			1.11	1.07E-10		
TEA030821.1	Aquaporin	1.13	0.0087407				
TEA002875.1	as a sensor for the physiological iron status within the plant, and or might be involved in the transport of iron	1.96	1.532E-05	2.07	2.12E-06		

TEA020761.1	as a sensor for the physiological iron status within the plant, and or might be involved in the transport of iron	2.19	0.0196858	2.15	0.0288388		
TEA002811.1	bidirectional sugar transporter					1.85	0.024644567
TEA022336.1	bidirectional sugar transporter			1.30	0.0190363		
TEA012371.1	C4-dicarboxylate transporter malic acid transport protein					-1.11	0.001230995
TEA024734.1	copper transporter	1.80	0.0005032				
TEA001794.1	equilibrative nucleoside transporter	-1.29	2.087E-05				
TEA013170.1	glucose-6-phosphate phosphate translocator	1.97	2.551E-05	1.77	0.0004958		
TEA018010.1	glycerol-3-phosphate transporter			1.31	1.21E-07		
TEA028265.1	inositol transporter			-1.80	0.008822		
TEA005381.1	K efflux antiporter 2	-5.79	0.0007648	-6.77	9.89E-05		
TEA019293.1	lysine histidine transporter			1.33	0.0203363		
TEA021874.1	Mitochondrial 2-oxoglutarate malate carrier			1.49	0.015848		
TEA021880.1	Mitochondrial 2-oxoglutarate malate carrier			1.68	3.76E-07	-1.68	5.54188E-07
TEA021881.1	Mitochondrial 2-oxoglutarate malate carrier			1.78	1.02E-08	-1.66	1.40784E-08
TEA031621.1	Mitochondrial 2-oxoglutarate malate carrier			1.74	1.21E-15	-2.30	2.11425E-26
TEA007748.1	mitochondrial substrate carrier family protein			-2.70	0.0384476		
TEA011526.1	Multidrug and toxin extrusion protein	-3.20	2.493E-19			-2.78	5.54789E-07
TEA033245.1	Multidrug and toxin extrusion protein			1.56	0.0018011	-1.11	0.027810135
TEA028217.1	Nitrate transporter					1.19	3.96925E-09
TEA007493.1	nitrite transporter	1.41	2.981E-10				
TEA030770.1	nitrite transporter					1.39	0.002171084
TEA020595.1	oligopeptide transporter	-1.08	1.778E-11				
TEA020606.1	oligopeptide transporter	-1.04	1.785E-07				
TEA012189.1	peptide nitrate transporter	1.56	0.0004257				
TEA031425.1	Peptide nitrate transporter					1.67	0.006647033
TEA024100.1	peptide transporter	-1.02	0.0003726				
TEA026555.1	peptide transporter					1.05	1.56786E-16
TEA016631.1	peptide transporter At1g52190-like	1.66	1.309E-05	1.14	0.0240457		

TEA032753.1	peptidyl-prolyl cis-trans isomerase	2.22	0.0353021				
TEA008904.1	plasma membrane intrinsic protein	1.25	0.002264911	1.85	7.90E-07		
TEA009357.1	plasma membrane intrinsic protein			1.57	6.48E-15	-1.06	5.23753E-06
TEA009696.1	plasma membrane intrinsic protein			1.26	0.0051904		
TEA019674.1	plasma membrane intrinsic protein			1.92	2.17E-06	-1.17	0.004063598
TEA005106.1	Pleiotropic drug resistance protein			1.22	7.25E-05		
TEA011180.1	Pleiotropic drug resistance protein	-6.26	7.95836E-11	-7.14	8.37E-10		
TEA022673.1	Pleiotropic drug resistance protein	-3.41	2.56566E-11	-2.84	3.31E-09		
TEA031848.1	Pleiotropic drug resistance protein	-1.19	4.1803E-05				
TEA028805.1	Potassium channel	-2.22	0.0483817				
TEA000803.1	SEC1 family transport protein	-1.27	0.0006845				
TEA014979.1	Sugar carrier protein					1.02	2.43015E-14
TEA002219.1	sugar transport protein			1.22	1.22E-15		
TEA015638.1	sulfate transporter			1.70	0.0059931		
TEA024313.1	Sulfite exporter TauE/SafE					-1.78	0.020222285
TEA024317.1	Sulfite exporter TauE/SafE			1.02	4.68E-12	-1.15	1.55597E-13
TEA019769.1	transmembrane protein 189-like					1.05	0.048273245
TEA017807.1	transporter	-1.80	0.0027984			-2.58	3.27822E-05
TEA028928.1	zinc transporter	2.33	4.332E-21	2.97	1.64E-37		

FC, fold change; FDR, false discovery rate.

Table S6. DEGs related to transcription factors.

GeneID	Description	L4 VS L1		L0 VS L1		L4 VS L0	
		**log2(FC)**	**FDR**	**log2(FC)**	**FDR**	**log2(FC)**	**FDR**
TEA019152.1	BZip transcription factor			2.39	0.0143214	-2.46	0.0131329
TEA005565.1	dof zinc finger protein	1.17	2.503E-07			1.35	1.262E-08
TEA016325.1	dof zinc finger protein			1.13	1.01E-12		
TEA017529.1	dof zinc finger protein	1.22	0.0046521				
TEA021053.1	dof zinc finger protein	1.36	3.916E-21			1.31	5.459E-17
TEA002675.1	E2F transcription factor-like E2FE-like			-2.19	0.0332807		
TEA023493.1	ethylene-responsive transcription factor 5-like	-1.31	1.273E-10			-2.12	6.294E-26
TEA009409.1	gata transcription factor	-1.78	0.030722				
TEA016123.1	GATA transcription factor			-1.12	0.0091559		
TEA018911.1	growth-regulating factor			-2.75	0.0004895	2.05	0.0405849
TEA024696.1	growth-regulating factor					1.16	0.0254249
TEA027740.1	growth-regulating factor			-4.80	0.0023145		
TEA023633.1	heat shock transcription factor	-1.39	3.689E-05			-1.54	5.509E-06
TEA005927.1	heat stress transcription factor	-1.19	1.069E-07				
TEA012764.1	heat stress transcription factor			1.06	1.04E-07	-1.83	7.112E-16
TEA004722.1	homeobox-leucine zipper protein	-2.46	2.323E-05	-1.42	0.0101038		
TEA009343.1	homeobox-leucine zipper protein	-1.18	6.978E-07				
TEA018436.1	homeobox-leucine zipper protein					1.32	1.103E-08
TEA019723.1	homeobox-leucine zipper protein			1.53	3.12E-05	-1.78	6.379E-08
TEA029286.1	homeobox-leucine zipper protein			-1.14	0.0026074		
TEA006275.1	Myb family transcription factor	-1.32	3.154E-09				
TEA026529.1	MYB family transcription factor	1.17	1.107E-15			1.01	4.041E-11
TEA011367.1	Myb transcription factor					-1.23	1.608E-20
TEA018025.1	Myb transcription factor					-1.05	0.0206983
TEA006889.1	Myb-like DNA-binding domain			1.38	1.42E-19	-1.08	1.061E-13

TEA014308.1	Myb-like DNA-binding domain	1.16	0.0013545	1.37	0.0047518		
TEA014310.1	Myb-like DNA-binding domain	1.58	1.07E-05	1.83	1.67E-06		
TEA014311.1	Myb-like DNA-binding domain	2.06	6.625E-14	2.29	0.0006906		
TEA022644.1	Myb-like DNA-binding domain			-2.25	0.0119897		
TEA029352.1	Myb-like DNA-binding domain			-1.50	0.0059283	1.62	0.0042639
TEA031815.1	Myb-like DNA-binding domain			1.10	0.0118074		
TEA031375.1	myb-like DNA-binding domain containing protein	1.59	2.451E-10	1.26	0.0001291		
TEA008294.1	Myb-related protein			1.84	0.0048626		
TEA029605.1	Myb-related protein	-1.08	0.0308784				
TEA033593.1	myb-related protein Myb4-like			1.16	0.0010058	-1.45	1.422E-06
TEA004609.1	nac domain			1.38	0.0097836		
TEA025924.1	nac domain			1.26	0.0018166	-1.95	1.733E-07
TEA025990.1	NAC domain containing protein	1.44	1.334E-05				
TEA015555.1	NAC domain-containing protein			2.88	3.86E-08	-3.63	8.217E-15
TEA018511.1	Ocs element-binding factor					-1.21	0.0225914
TEA031366.1	Ocs element-binding factor	1.83	2.838E-13				
TEA027043.1	Peptide chain release factor	-1.44	0.02972				
TEA002059.1	transcription factor BEE	1.25	0.0015532	-1.57	0.0177082	2.85	2.383E-12
TEA013107.1	transcription factor BEE					2.35	0.0072606
TEA024004.1	transcription factor bHLH25-like	1.25	0.0214578	1.68	0.000815		
TEA021575.1	transcription factor jumonji (jmjC) domain-containing protein			-3.16	0.0159485		
TEA007980.1	Transcription initiation factor TFIID	-3.64	0.0085588	-2.46	0.0438286		
TEA005853.1	Transcriptional activator	-1.24	0.0096067				
TEA027058.1	WRKY	1.16	2.623E-14	2.03	1.05E-08		
TEA007370.1	WRKY transcription factor			1.12	2.96E-11		
TEA029100.1	WRKY transcription factor	-1.66	1.921E-11			-1.71	1.589E-06
TEA000134.1	zinc finger			1.24	1.91E-05		
TEA003400.1	zinc finger			1.23	2.68E-11	-1.22	6.444E-11

TEA003799.1	zinc finger			2.32	1.72E-48	-3.18	1.134E-90
TEA010800.1	zinc finger	-1.55	1.005E-15			-1.05	8.369E-05
TEA013686.1	zinc finger AN1 domain-containing stress-associated protein			1.50	0.0030376	-1.70	3.478E-05
TEA003220.1	zinc finger CCCH domain-containing protein					-1.13	4.281E-15
TEA012218.1	zinc finger CCCH domain-containing protein	-2.44	0.0026869	-2.06	0.0189626		
TEA014464.1	Zinc finger protein	-2.22	0.0174511				
TEA016175.1	Zinc finger protein	-1.27	0.0022978				
TEA018181.1	Zinc finger protein			1.64	4.45E-17	-2.27	5.161E-49
TEA029028.1	Zinc finger protein			1.41	8.20E-06	-1.88	4.518E-13
TEA030850.1	Zinc finger protein			1.17	3.55E-06	-1.48	1.765E-09
TEA031339.1	Zinc finger protein	-1.26	0.0011815			-2.07	1.643E-12
TEA033694.1	Zinc finger protein			-1.16	0.0375332		
TEA004343.1	Zinc finger protein CONSTANS-LIKE			1.20	3.33E-16		
TEA018780.1	Zinc finger protein CONSTANS-LIKE	2.02	3.758E-22			1.50	2.026E-14
TEA024080.1	Zinc finger, C3HC4 type (RING finger)	-1.84	0.0462558				
TEA020473.1	ZnF_C2H2			3.02	8.94E-58	-3.66	1.878E-67
TEA005626.1	ZnF_C3H1	-1.10	0.0093999				

FC, fold change; FDR, false discovery rate.

Table S7. DEGs related to signal transduction.

geneID	Description	L4 VS L1		L0 VS L1		L4 VS L0	
		log2(FC)	FDR	log2(FC)	FDR	log2(FC)	FDR
Protein phosphorylation							
TEA016698.1	Cbl-interacting protein kinase					-1.12	0.0013995
TEA012649.1	glutamate receptor	-1.08	3.418E-07			-1.19	0.0001758
TEA015966.1	glutamate receptor			1.55	0.0451905		
TEA006325.1	glutamate-gated kainate-type ion channel receptor subunit GluR5	-1.57	0.0268243				
TEA022399.1	Glutamate-gated receptor that probably acts as non- selective cation channel (By similarity)	-1.13	0.0187992				
TEA003693.1	G-type lectin S-receptor-like serine threonine-protein kinase					-1.44	1.029E-06
TEA014297.1	G-type lectin S-receptor-like serine threonine-protein kinase	-1.52	7.458E-08	-4.24	2.449E-28	2.73	8.402E-10
TEA016133.1	G-type lectin S-receptor-like serine threonine-protein kinase	1.82	0.0266382				
TEA023653.1	G-type lectin S-receptor-like serine threonine-protein kinase	-1.89	1.613E-10	-1.40	1.889E-06		
TEA004198.1	G-type lectin S-receptor-like serine threonine-protein kinase RLK1-like	-7.90	5.17E-08	-7.92	5.976E-08		
TEA026954.1	His Kinase A (phospho-acceptor) domain					-1.15	0.0481707
TEA029300.1	Inactive receptor kinase			1.53	0.0006876	-1.46	0.0001868
TEA008087.1	Inherit from KOG: A kinase (PRKA) anchor protein					-1.25	1.754E-05
TEA005925.1	Late embryogenesis abundant protein			1.50	3.992E-07		
TEA030980.1	Late embryogenesis abundant protein			1.32	0.0013505		
TEA004734.1	Lectin-domain containing receptor kinase			-1.94	4.076E-05		
TEA001285.1	Leucine rich repeat N-terminal domain	-2.66	0.0002381				
TEA005463.1	Leucine zipper-ef-hand containing transmembrane protein	-1.23	4.824E-06				
TEA010346.1	Leucine-rich repeat receptor-like protein kinase	-1.11	5.398E-10				
TEA010364.1	Leucine-rich repeat receptor-like protein kinase	-1.65	1.588E-14	-1.04	2.863E-06		
TEA000685.1	LRR receptor-like serine threonine-protein kinase	-1.05	0.0234619	-1.35	0.0038029		

TEA001426.1	LRR receptor-like serine threonine-protein kinase	-1.18	0.0240572				
TEA008110.1	LRR receptor-like serine threonine-protein kinase			-4.35	0.0139382		
TEA008725.1	LRR receptor-like serine threonine-protein kinase	-1.03	5.175E-05				
TEA009342.1	LRR receptor-like serine threonine-protein kinase			1.13	6.168E-06		
TEA019444.1	LRR receptor-like serine threonine-protein kinase	-1.23	0.00017	-1.10	0.0004196		
TEA020091.1	LRR receptor-like serine threonine-protein kinase	-1.40	0.0011977	-1.34	0.0028729		
TEA020829.1	LRR receptor-like serine threonine-protein kinase			-2.45	0.0123476		
TEA022995.1	LRR receptor-like serine threonine-protein kinase	-1.24	3.509E-05				
TEA024023.1	LRR receptor-like serine threonine-protein kinase	-1.19	3.528E-05				
TEA026048.1	LRR receptor-like serine threonine-protein kinase			1.12	0.0007964		
TEA028616.1	LRR receptor-like serine threonine-protein kinase			1.87	0.0359452		
TEA031252.1	LRR receptor-like serine threonine-protein kinase	-1.26	9.461E-06				
TEA031361.1	LRR receptor-like serine threonine-protein kinase	-1.84	0.0441636				
TEA032402.1	LRR receptor-like serine threonine-protein kinase	-2.09	0.0334967				
TEA033409.1	LRR receptor-like serine threonine-protein kinase	-1.73	0.0247203				
TEA009028.1	LRR receptor-like serine threonine-protein kinase At4g08850-like	-1.96	2.191E-07				
TEA019012.1	L-type lectin-domain containing receptor kinase S.7-like			-1.58	7.632E-05		
TEA018931.1	membrane-associated kinase regulator 2-like	1.61	2.202E-06	1.35	0.0006028		
TEA021093.1	membrane-associated kinase regulator 2-like			-1.22	0.0444354		
TEA027823.1	nodulation-signaling pathway 2 protein-like	1.02	0.0375505	1.86	8.7E-05		
TEA002802.1	probably inactive leucine-rich repeat receptor-like protein kinase					-1.23	5.332E-10
TEA004731.1	receptor kinase			2.40	0.0165071		
TEA008707.1	receptor kinase	-1.01	0.0018902				
TEA023128.1	Receptor like protein	-7.17	2.253E-08	-9.05	4.531E-06		
TEA027621.1	receptor like protein 27	-1.42	2.296E-06			-1.23	0.00085
TEA003563.1	receptor protein kinase	-1.99	0.0139453	-2.07	0.0022947		
TEA023569.1	receptor protein kinase	-1.23	0.0003727				

TEA024197.1	receptor protein kinase TMK1-like					-1.41	0.0001508
TEA023077.1	receptor-like protein	1.49	0.0261982	2.15	0.000854		
TEA004458.1	receptor-like protein 12-like			-1.85	0.002327		
TEA018249.1	receptor-like protein 12-like			1.12	0.0009705		
TEA019693.1	receptor-like protein 12-like	-1.05	0.0013562				
TEA021436.1	receptor-like protein 12-like	-1.21	0.0083818	-1.05	0.0368992		
TEA022736.1	receptor-like protein 12-like					-1.27	0.0224435
TEA001173.1	receptor-like protein kinase			-3.97	0.0003907		
TEA002378.1	receptor-like protein kinase			-1.16	0.0109776		
TEA003742.1	receptor-like protein kinase					-1.05	0.0003444
TEA003887.1	receptor-like protein kinase					-1.07	1.059E-08
TEA004467.1	receptor-like protein kinase	-1.95	0.001871	-1.63	0.0172579		
TEA005802.1	receptor-like protein kinase					-1.25	3.212E-05
TEA007751.1	receptor-like protein kinase	-1.29	0.0024198				
TEA011072.1	receptor-like protein kinase	1.06	2.904E-05	1.89	2.611E-10		
TEA011689.1	receptor-like protein kinase					1.82	0.0161663
TEA021752.1	receptor-like protein kinase					-1.24	5.553E-05
TEA024202.1	receptor-like protein kinase					-1.10	0.0130308
TEA024920.1	receptor-like protein kinase	-1.17	3.607E-11			-1.29	3.05E-08
TEA025731.1	receptor-like protein kinase					-1.09	4.951E-09
TEA027016.1	receptor-like protein kinase	-1.09	7.869E-05			-1.77	2.277E-06
TEA032368.1	receptor-like protein kinase	-1.83	0.0050653				
TEA002699.1	receptor-like protein kinase precursor			-1.95	0.0146796		
TEA008074.1	receptor-like protein kinase precursor	-6.98	0.0079726	-7.01	8.031E-05		
TEA033339.1	receptor-like serine threonine-protein kinase ALE2-like	-2.15	8.794E-06	-3.17	4.513E-09		
TEA002002.1	Serine Threonine protein kinase					-1.07	8.705E-11
TEA015937.1	Serine Threonine protein kinase	-1.50	0.010818	-1.90	0.0015231		
TEA000578.1	serine threonine-protein kinase	2.39	0.0427643				
TEA001010.1	Serine threonine-protein kinase					-1.28	1.403E-10

TEA002760.1	Serine threonine-protein kinase	-1.95	5.779E-11	-1.28	9.339E-06		
TEA002897.1	serine threonine-protein kinase	-1.16	0.0416872				
TEA004552.1	Serine threonine-protein kinase					-1.08	0.0328029
TEA007346.1	Serine threonine-protein kinase					-1.07	2.754E-05
TEA010212.1	serine threonine-protein kinase					1.61	0.0417815
TEA010523.1	Serine threonine-protein kinase					-1.10	0.0296365
TEA011133.1	serine threonine-protein kinase	-2.71	5.909E-06	-2.56	6.824E-05		
TEA019497.1	Serine threonine-protein kinase	-1.05	0.0063273				
TEA020499.1	serine threonine-protein kinase					-1.34	6.866E-09
TEA021086.1	Serine threonine-protein kinase					-1.01	0.0001074
TEA024251.1	Serine threonine-protein kinase			-1.06	0.0156475		
TEA025402.1	serine threonine-protein kinase	1.72	0.012269				
TEA025623.1	serine threonine-protein kinase	-1.17	0.0283448				
TEA025888.1	serine threonine-protein kinase	1.43	0.0031807				
TEA028331.1	serine threonine-protein kinase					1.04	0.0007633
TEA029625.1	Serine threonine-protein kinase	-1.38	9.26E-07	-1.02	0.0007513		
TEA029637.1	serine threonine-protein kinase	-1.91	0.0106933				
TEA029684.1	Serine threonine-protein kinase	-1.12	0.0368631			-1.36	0.0117226
TEA031442.1	Serine threonine-protein kinase	-2.09	1.076E-08	-1.75	6.678E-06		
TEA010756.1	Serine threonine-protein kinase-like protein					-1.06	8.722E-06
TEA033674.1	Serine threonine-protein kinase-like protein	-1.59	7.006E-12			-2.49	4.836E-17
TEA018455.1	serine threonine-protein phosphatase 2A 65 kDa regulatory subunit A	-1.87	0.0130914				
TEA023848.1	Signal peptidase, peptidase S26	-1.16	0.0085585				
TEA021185.1	Somatic embryogenesis receptor kinase					1.01	0.002469
TEA013049.1	Wall-associated receptor kinase	-6.67	8.038E-06	-5.26	4.486E-06		
TEA020039.1	Wall-associated receptor kinase	-2.19	0.0006047				
TEA024220.1	Wall-associated receptor kinase			1.87	0.0293581		
Protein dephosphorylation							

TEA031977.1	Endonuclease Exonuclease phosphatase family protein	-1.26	7.656E-08				
TEA005633.1	Multiple inositol polyphosphate phosphatase	-1.64	2.46E-13	-1.43	5.193E-11		
TEA015199.1	phosphatase 2C	-1.07	3.37E-10			-1.21	5.956E-06
TEA017117.1	phosphatase 2C			2.16	0.0001816	-3.03	6.874E-08
TEA026107.1	phosphatase 2C			-2.61	0.0344498		
TEA026957.1	phosphatase 2C					-1.15	1.245E-07
TEA029597.1	phosphatase 2C	-1.29	2.555E-18	1.16	7.245E-07	-2.44	1.581E-27
TEA033105.1	Protein phosphatase 1 regulatory subunit			-1.07	0.0005639		
TEA026994.1	protein phosphatase 2C 6-like	1.21	1.65E-13				
TEA003380.1	protein-tyrosine-phosphatase			5.23	0.0029239		
TEA000708.1	Purple acid phosphatase					-1.32	0.0261875
TEA019918.1	Purple acid phosphatase			1.28	5.399E-05		
TEA032835.1	Purple acid phosphatase	1.15	4.215E-09				
TEA018455.1	serine threonine-protein phosphatase 2A 65 kDa regulatory subunit A	-1.87	0.0130914				
TEA010268.1	type I inositol-1,4,5-trisphosphate 5-phosphatase	1.26	0.0342937				
hormone-mediated signal							
TEA013231.1	1-aminocyclopropane-1-carboxylate			-1.47	0.0495183		
TEA010264.1	abscisate beta-glucosyltransferase-like	2.69	2.731E-27	2.23	2.066E-14		
TEA024137.1	abscisate beta-glucosyltransferase-like	1.89	5.541E-05	1.74	0.0002691		
TEA014723.1	Abscisic acid 8'-hydroxylase	-1.70	1.935E-08			-1.76	2.529E-05
TEA031407.1	Abscisic acid 8'-hydroxylase	-1.64	0.0040697				
TEA010094.1	abscisic acid receptor					1.65	0.0355212
TEA016225.1	abscisic acid receptor			-1.20	8.236E-08	1.09	3.893E-06
TEA027409.1	Abscisic acid-insensitive 5-like protein	1.49	0.0159901				
TEA006724.1	AUX/IAA family					1.09	8.211E-15
TEA006764.1	auxin efflux carrier			1.62	0.0309977		
TEA007905.1	auxin response factor	-1.82	0.0043374	-1.71	0.0070169		
TEA021183.1	Auxin responsive protein					1.37	0.0162746

TEA033527.1	Auxin responsive protein			2.29	1.467E-09	-2.22	2.529E-10
TEA003908.1	auxin transporter-like protein					1.17	9.322E-06
TEA009090.1	auxin transporter-like protein			1.07	0.0041079		
TEA005097.1	auxin-induced protein			1.27	0.0255651		
TEA029993.1	Auxin-induced protein			-1.47	0.0315413		
TEA030078.1	auxin-responsive					-1.22	5.533E-05
TEA006379.1	auxin-responsive protein			1.01	2.878E-08		
TEA009753.1	Cis-zeatin O-glucosyltransferase					3.14	0.0006406
TEA015118.1	cysteine histidine-rich C1 domain-containing protein					2.32	0.0005786
TEA031174.1	Cysteine-rich receptor-like protein kinase					4.40	9.766E-35
TEA020986.1	cytokinin	1.49	6.484E-09	2.22	0.000416		
TEA023162.1	cytokinin riboside 5'-monophosphate phosphoribohydrolase			1.40	0.0074986	-1.67	0.0008667
TEA033408.1	Dormancy/auxin associated protein			1.01	4.958E-12		
TEA008202.1	EF hand family protein			1.11	0.004128		
TEA014324.1	EF hand family protein			1.44	1.461E-09		
TEA021043.1	EF hand family protein	-1.28	6.376E-07	1.95	8.042E-24		
TEA012689.1	EH domain-containing protein	-1.18	2.346E-05				
TEA023142.1	EID1-like F-box protein	-2.52	2.07E-37	-1.08	3.565E-07		
TEA004320.1	EIN3-binding F-box protein			-2.76	4.096E-57	3.36	4.28E-133
TEA006639.1	EIN3-binding F-box protein					1.11	4.718E-15
TEA028789.1	EIN3-binding F-box protein					1.66	6.715E-27
TEA002824.1	Ethylene receptor	1.27	7.245E-22			1.65	7.716E-32
TEA020178.1	Ethylene receptor	1.59	9.75E-33			2.34	5.7E-43
TEA026952.1	Ethylene receptor	2.23	0.0062303			3.15	0.0005458
TEA014251.1	gibberellin			-1.43	0.0006562	2.27	1.313E-12
TEA024383.1	gibberellin	-2.09	0.0001907	-1.28	0.038504		
TEA023946.1	gibberellin 20 oxidase	1.07	2.46E-13				
TEA023946.1	gibberellin 20 oxidase			1.47	9.297E-19		
TEA001361.1	gibberellin 3-beta-dioxygenase	1.68	0.0063011				

TEA013731.1	Indole-3-acetic acid-amido synthetase			1.33	0.0251527	-2.00	8.744E-06
TEA020186.1	Indole-3-acetic acid-amido synthetase			1.20	0.0354765		
TEA031000.1	Inherit from euNOG: Ethylene-responsive nuclear protein			1.32	5.299E-13	-1.94	8.879E-27
Ca calmodulin-mediated signal							
TEA008626.1	ATPase, Ca transporting, plasma membrane					-1.64	0.0036186
TEA014010.1	calcineurin b-like protein	-1.07	0.000388				
TEA032298.1	Calcineurin subunit	1.03	0.0032038				
TEA007881.1	calcium-binding protein			3.39	1.373E-07	-4.17	8.151E-10
TEA018321.1	calcium-binding protein			1.00	5.169E-08	-1.14	6.884E-09
TEA023001.1	calcium-binding protein			1.44	0.0103324	-1.65	0.002193
TEA025822.1	calcium-binding protein			2.51	0.0007442	-2.56	0.0013807
TEA030247.1	calcium-binding protein	1.04	0.0008828	1.05	0.003543		
TEA022753.1	calcium-transporting ATPase					-1.16	0.0417815
TEA032463.1	Calcium-transporting ATPase			2.65	0.0391035		
TEA007785.1	calcium-transporting ATPase 3, endoplasmic	-1.18	0.0048279				
TEA017804.1	calcium-transporting ATPase 3, endoplasmic	-1.20	0.0281176	-1.30	0.0122255		
TEA033555.1	calcium-transporting ATPase 3, endoplasmic	-1.21	4.636E-12				
TEA033561.1	calcium-transporting ATPase 3, endoplasmic	-1.12	0.0004062				
TEA014836.1	Calmodulin binding protein-like	-1.12	0.0048574				
TEA009049.1	Inherit from NOG: calmodulin-binding family					-1.32	4.562E-06
TEA008476.1	IQ calmodulin-binding motif domain containing protein, expressed					-1.32	0.0004234
TEA011080.1	IQ calmodulin-binding motif family protein					1.09	0.0007187
TEA027920.1	sodium calcium exchanger family protein			1.37	0.0147524		
TEA014609.1	Sodium channel voltage-gated type	-1.04	0.0013862				
TEA006137.1	Sodium/calcium exchanger protein	1.16	0.0095186				
TEA021659.1	Sodium/calcium exchanger protein			1.05	7.444E-09	-1.00	1.285E-07
TEA033083.1	Sodium/calcium exchanger protein	-1.94	0.0090184	-6.13	1.71E-05		

FC, fold change; FDR, false discovery rate.

Table S8. DEGs related to phenolics.

geneID	Description	L4 VS L1		L0 VS L1		L4 VS L0	
		log2(FC)	FDR	log2(FC)	FDR	log2(FC)	FDR
**TEA034012.1**	**4-coumarate--CoA ligase**			**1.12**	**7.129E-07**		
**TEA009266.1**	**anthocyanidin reductase**	**1.19**	**1.382E-16**	**1.04**	**0.0331177**		
**TEA022960.1**	**anthocyanidin reductase**	**1.07**	**2.048E-14**	**1.41**	**0.0001273**		
**TEA010322.1**	**anthocyanidin synthase**	**2.31**	**4.224E-16**	**3.11**	**0.0005394**		
TEA000809.1	Arogenate dehydratase prephenate dehydratase			1.68	1.44E-15	-1.51	2.716E-12
TEA001026.1	Arogenate dehydratase prephenate dehydratase			1.26	5.609E-05		
**TEA002954.1**	**bifunctional dihydroflavonol 4-reductase flavanone**	**1.40**	**2.406E-07**				
TEA016966.1	cyanidin-3-O-glucoside 2-O-glucuronosyltransferase-like	1.09	0.0195909				
TEA008672.1	dehydratase shikimate dehydrogenase			1.41	5.205E-07		
**TEA032730.1**	**Dihydroflavonol4-reductase**			**1.79**	**0.0035176**		
**TEA023790.1**	**flavanone 3-hydroxylase**	**1.86**	**3.04E-39**	**1.88**	**0.0003848**		
TEA006847.1	flavonoid	1.84	2.716E-06	2.02	0.0041287		
TEA014249.1	flavonol 3-O-glucosyltransferase activity	2.50	5.924E-30	1.81	3.508E-10		
TEA014260.1	flavonol 3-O-glucosyltransferase activity	2.11	8.45E-27	1.63	6.968E-13		
TEA028622.1	Flavonol synthase flavanone	-1.42	0.027206				
**TEA006643.1**	**flavonol synthase flavanone 3-hydroxylase-like**	**1.37**	**2.246E-13**	**1.85**	**5.337E-05**		
TEA030910.1	hydroxycinnamoyl-Coenzyme A shikimate quinate hydroxycinnamoyltransferase-like	-5.88	5.251E-08	-4.74	2.108E-08		
TEA015167.1	Isoflavone reductase	1.38	0.0263663			1.00	0.0395354
TEA015375.1	Isoflavone reductase	1.15	7.338E-13				
TEA027426.1	Isoflavone reductase					1.09	1.767E-10
**TEA027582.1**	**Leucoanthocyanidin reductase**	**2.41**	**2.06E-05**	**2.67**	**0.0019755**		
**TEA003137.1**	**Phenylalanine ammonialyase**	**1.40**	**5.269E-10**	**2.07**	**0.0028849**		
**TEA003374.1**	**Phenylalanine ammonialyase**			**1.07**	**3.777E-08**		
**TEA014056.1**	**Phenylalanine ammonialyase**			**1.02**	**3.65E-06**		

**TEA023243.1**	**Phenylalanine ammonialyase**	**1.91**	**3.037E-22**	**2.79**	**1.909E-05**		
**TEA024587.1**	**Phenylalanine ammonialyase**			**1.38**	**0.0031966**		
**TEA034008.1**	**Phenylalanine ammonialyase**			**1.39**	**0.0062817**		
TEA023331.1	The primary product of this enzyme is 4,2',4',6'-tetrahydroxychalcone					2.45	2.745E-08
TEA014864.1	trans-cinnamate			1.04	1.988E-11		
TEA034001.1	trans-cinnamate			1.91	0.0041657		

FC, fold change; FDR, false discovery rate. DEGs related to catechin biosynthesis were highlighted in bold.

Table S9. Specific primer pairs used for qRT-PCR expression analysis.

NO.	Genes	Accession No.	Forward primers (5′→3′)	Reverse primers (5′→3′)
A	flavanone 3-hydroxylase	TEA023790.1	CCAACAACAACGCTTACGGCTCTC	ACCTTCACATGCCTCCACAATCTTCC
B	Auxin responsive protein	TEA011595.1	ACCAAACGCATCTCCAGGCTATTCAG	CCACCGCTTTGTCTTCTTCCCATAGAG
C	Galactinol--sucrose galactosyltransferase	TEA013682.1	GCCTCCATTACTCGGAACTTCCAAGAC	TGTACGCAACTGATGAGATGTGGATGG
D	AAA-type ATPase family protein	TEA022839.1	AAGCCTACACTGCCATTGAACGGTAC	TCCTTGAAGCCCACTTGAGTTTGATCC
E	anthocyanidin reductase	TEA022960.1	AATGCTGTCAGGCTCAATCTCCATCTC	GCCGATTCTTTCTCTGCCACAAACAC
F	Mitochondrial 2-oxoglutarate malate carrier	TEA021874.1	ACCGCATCACAGCTCGCATCATAC	CCAGGCTCCACCTTCATGTTCATAACC
G	calcium-binding protein	TEA018321.1	ACGACACGTTCTTCGGATTCGATGATG	ATCTGATTCCTCTTGCTTCGCCTTCG
H	CCR4-associated factor	TEA012350.1	TCACGGTTGTCGGATGAGGCTACT	TCGGAGAGCGTAAGACCTAACTGGAT
I	Aquaporin	TEA019952.1	AGGAAGGGCGAGTTGGGAATTATAGC	CGCAGCCAATGCACTACCAATAAGAG
J	Mitochondrial 2-oxoglutarate malate carrier	TEA031621.1	GCAGTTACAAGGCGAGAACCATGT	AATCTTGATTCCGACGGCGATGG
K	Glutathione S-transferase	TEA000598.1	GGTTATGGAAGAGGAGGATCGCAATCG	TGCTCCCAACAACCGCTGCTTT
L	EF hand family protein	TEA014324.1	TCGAAGAAGATGGACATCTACGCAGAG	AACCCAACCTCTCCTGAAGTTTCTCAC
